# Chloroplast primers for clade‐wide phylogenetic studies of *Thalictrum*

**DOI:** 10.1002/aps3.11294

**Published:** 2019-10-16

**Authors:** Diego F. Morales‐Briones, Tatiana Arias, Verónica S. Di Stilio, David C. Tank

**Affiliations:** ^1^ Department of Biological Sciences University of Idaho 875 Perimeter Dr. MS 3051 Moscow Idaho 83844‐3051 USA; ^2^ Stillinger Herbarium University of Idaho 875 Perimeter Dr. MS 3026 Moscow Idaho 83844-3026 USA; ^3^ Institute for Bioinformatics and Evolutionary Studies (IBEST) University of Idaho 875 Perimeter Dr. MS 3051 Moscow Idaho 83844‐3051 USA; ^4^ School of Biological Sciences The University of Hong Kong Pokfulam Road Hong Kong Hong Kong; ^5^ Corporación para Investigaciones Biológicas Cra. 72 A No. 78 B 141 Medellín Colombia; ^6^ Department of Biology University of Washington Box 351800 Seattle Washington 98195‐1800 USA; ^7^ Present address: Department of Plant and Microbial Biology University of Minnesota 1479 Gortner Avenue Saint Paul Minnesota 55108‐1095 USA

**Keywords:** chloroplast genome, high‐throughput sequencing, meadow‐rue, microfluidic PCR, Ranunculaceae, *Thalictrum thalictroides*

## Abstract

**Premise:**

Chloroplast primers were developed for phylogenetic and comparative studies in *Thalictrum* (Ranunculaceae).

**Methods and Results:**

We assembled and annotated the complete plastome sequence of *T. thalictroides* by combining multiple whole genome sequencing libraries. Using transcriptome‐sequencing libraries, we also assembled a partial plastome of the related species *T. hernandezii*. From the newly assembled plastomes and one previously sequenced plastome, we designed and validated 28 primer pairs to target variable portions of the chloroplast genome in *Thalictrum*. Furthermore, we tested the validated primers in 62 species of *Thalictrum*. The total alignment length of the 28 regions was 15,268 bp with 2443 variable sites and 92% character occupancy.

**Conclusions:**

The newly developed chloroplast primer pairs improve the phylogenetic resolution (bootstrap support and tree certainty) in *Thalictum* and will be a useful resource for future phylogenetic and evolutionary studies for species in the genus and in close relatives in Thalictroideae.

The chloroplast genome (cpDNA) has been particularly useful for resolving evolutionary relationships in plants for the past 30 years (reviewed in Gitzendanner et al., 2018). High‐throughput sequencing has facilitated the development of various approaches for collecting multiple regions or complete sequences of this genome (reviewed in Twyford and Ness, 2016). Furthermore, approaches based on PCR target enrichment in combination with high‐throughput sequencing (e.g., Uribe‐Convers et al., 2016) have proven to be a cost‐effective approach for sequencing multiple chloroplast regions simultaneously, and have been successfully applied in phylogenetic studies (e.g., Jacobs et al., 2018; Morales‐Briones and Tank, 2019).

*Thalictrum* L. (Thalictroideae, Ranunculaceae) is a clade of ca. 190 species of herbaceous perennials distributed primarily in northern temperate regions (Tamura, 1995) with a diversity of sexual systems (hermaphroditic, dioecious, andromonoecious, or gynomonoecious [Boivin, 1944]), pollination mode (insect or wind [Soza et al., 2012; Wang et al., 2018]), and ploidy (from 2*x* =14 to 30*x* = 210 [Soza et al., 2013]). To date, molecular phylogenetic studies of *Thalictrum* have relied on sequences of the nuclear ribosomal DNA (nrDNA) cistron, especially the internal transcribed spacer (ITS) and external transcribed spacer (ETS) regions, and up to five cpDNA regions (Soza et al., 2012, 2013; Wang et al., 2018). Although Soza et al. (2013) surveyed several cpDNA regions from Shaw et al. (2007), only one was identified as sufficiently variable for phylogenetic analyses in *Thalictrum*. Here, we assembled and annotated the complete plastome of *T. thalictroides* (L.) A. J. Eames & B. Boivin, assembled a partial plastome of *T. hernandezii* Tausch ex J. Presl, and designed and validated PCR primers that target highly variable chloroplast regions in *Thalictrum* to aid in future phylogenetic studies of this group and close relatives.

## METHODS AND RESULTS

### Plastome assembly

Genomic libraries of *T. thalictroides* and transcriptome libraries of *T. hernandezii* were downloaded from the National Center for Biotechnology Information (NCBI) Sequence Read Archive (Appendix 1). Due to low cpDNA coverage in the transcriptome libraries, a reference‐guided assembly of *T. hernandezii* was carried out using Alignreads version 2.5.2 (Straub et al., 2011) with *T. coreanum* H. Lév. as a reference (Park et al., 2015), with one inverted repeat (IR) removed. We obtained a plastid consensus sequence made of 151 contigs representing 116,700 bp (after removal of regions not covered in the reference) for *T. hernandezii*. Given the availability of multiple genome sequencing libraries for two samples of *T. thalictroides* (Appendix 1), we performed de novo assemblies for this species. Assemblies were carried out for the two individuals of *T. thalictroides* (WT478 and WTBG) separately using the Fast‐Plast version 1.2.6 pipeline (McKain and Wilson, 2017). Single contigs representing the complete chloroplast genome of *T. thalictroides* were obtained. The resulting complete plastomes of *T. thalictroides* were annotated using CpGAVAS (Liu et al., 2012). Genes encoding for tRNAs were verified using tRNAscan‐SE version 2.0 (Lowe and Chan, 2016). Annotations were verified and edited in Geneious version 7.1.9 (Kearse et al., 2012) using other available Ranunculaceae plastomes as references (Appendix 1). The genome map was drawn with OGDraw version 1.2 (Lohse et al., 2013). Characterization of the *T. thalictroides* plastome and its comparison with the plastome of *T. coreanum* (Park et al., 2015) can be found in Appendix S1.

### Primer design

Plastome sequences of *T. thalictroides*,* T. hernandezii*, and *T. coreanum* (all with one IR removed) were aligned using MAFFT version 7.017b (Katoh and Standley, 2013). The most variable regions of the alignment, spanning 400–600 bp, were identified using a custom R script (Uribe‐Convers et al., 2016). Primer design was carried out using Primer3 (Untergasser et al., 2012) following the specifications of the microfluidic PCR Access Array system protocol (Fluidigm, San Francisco, California, USA), with an annealing temperature of 60°C (±1°C) and no more than three continuous nucleotides of the same base. Primer validation followed Uribe‐Convers et al. (2016) by simulating the four‐primer reaction of the microfluidic PCR. We used our target‐specific primers and 5′ conserved sequence (CS) tags to provide annealing sites for Illumina sequencing adapters and sample‐specific barcodes. PCR validations were done using genomic DNA from three *Thalictrum* species (Appendix 2) and a negative control that did not contain DNA. Amplicons were visualized in a QIAxcel Advanced System (QIAGEN, Valencia, California, USA) and scored following Uribe‐Convers et al. (2016).

A total of 81 primer pairs were designed, of which 28 passed the validation step (Table 1), 32 failed (i.e., failed to amplify in one or more species, produced significant primer dimers, and/or produced multiple amplicons), and 21 were not validated (Appendix S2). In order to test cross‐amplification of the 28 validated PCR primer pairs, we amplified and sequenced these regions on 75 individuals of *Thalictrum* representing 62 species from 13 of 14 described sections of the genus (Tamura, 1995). Our sampling represents 33% of *Thalictrum* species and 70% of the sampling used in the most recent molecular phylogeny of the genus (Wang et al., 2018). Additionally, we amplified the newly designed regions from one individual each of three related genera in Thalictroideae (*Aquilegia* L., *Leptopyrum* Rchb., and *Paraquilegia* J. R. Drumm. & Hutch.; Appendix 2) using previously extracted DNA samples by Soza et al. (2012). Microfluidic PCR was carried out on the Access Array system (Fluidigm) following the manufacturer's protocol. PCR amplicons were multiplexed, and sequenced in an Illumina MiSeq (San Diego, California, USA) with 300‐bp paired‐end reads. Raw reads were cleaned, demultiplexed, and merged using the dbcAmplicons pipeline (Uribe‐Convers et al., 2016). Consensus sequences for each sample in all amplicons were generated using the ‘reduce_amplicons’ R script (part of dbcAmplicons). Each chloroplast region was aligned with MAFFT and alignment summary statistics were calculated with AMAS (Borowiec, 2016).

**Table 1:** Sequence of validated primers for the *Thalictrum* chloroplast genome.^a^

Locus	Primer sequences (5′–3′)^b^	Amplification region	Chloroplast region	Amplicon length, bp^c^
thal‐13	F: GCAATAAGTCCGGTTTGCAT	*atpA*, (*atpA*‐*atpF*) IGS, *atpF*	LSC	550
	R: GGGCGATGAAAGAAATAAACG			
thal‐15	F: ACATGGCTTTCTTCCATAACG	(*atpH‐aptI*) IGS, *atpI*	LSC	574
	R: GAATCCATGGAGGGTCATCA			
thal‐44	F: TGAAGTGATAGCCCGATTCC	(*rbcL‐accD*) IGS, *accD*	LSC	518
	R: TTTCCAGTTCATTCCGATCA			
thal‐45	F: TGATGGGTCTAAGAGTGACAATCA	*accD*	LSC	419
	R: CGATTCTTTCTGAACTGCTCATT			
thal‐46	F: TTTGCAGCATTGAGTAAGGAAC	(*ycf4‐cemA*) IGS	LSC	577
	R: CCCGAACGAGTCATTTCAA			
thal‐47	F: GAGAAGGTTCAATTGTCCGAAA	*petA*, (*petA‐psbJ*) IGS, *psbJ*	LSC	572
	R: GGTATTCTTGTGATCGGTTTACTAGG			
thal‐50	F: TGAGGTGATTGGATTTGCAC	(*rpl20‐clpP*) IGS, *clpP*	LSC	558
	R: CGAAGACATGGAAAGGGATG			
thal‐51	F: AACCCTTGTGAGGGTTTCG	*clpP*	LSC	541
	R: GAGGCCTCTTTCCAATATTTATGTTA			
thal‐52	F: TTACATATTGCGAAGGCATAGTCT	*clpP*	LSC	414
	R: TGAACCGTATGCATCCAAAG			
thal‐53	F: AAGAATCAATGTGCTGATTCCA	*clpP*	LSC	534
	R: GTATCCAGGCTCCGTTCAGA			
thal‐54	F: TCTGAACGGAGCCTGGATAC	*clpP*, (*clpP‐psbB*) IGS	LSC	560
	R: TTCGTAGGAACAAAGATAAGCAGA			
thal‐55	F: TGCTCTTGTATCTTTCGCCTCT	(*psbB‐psbT*) IGS, *psbT*, (*psbT‐psbN*) IGS, *psbN*, (*psbN‐psbH*) IGS	LSC	525
	R: CATTGCGGTCTTGCAATTT			
thal‐57	F: CTGGCTCCGTAAGATCCAGT	*petD*	LSC	513
	R: CGAAGGAACCGGACATGATA			
thal‐58	F: GGAGCAACATTGCCTATTGATAA	*petD*, (*petD‐rpoA*) IGS, *rpoA*	LSC	546
	R: CAATCAAGGCAGGGTTACTTTAC			
thal‐59	F: TAACCCTGCCTTGATTGTCC	*rpoA*	LSC	565
	R: GGAACATGTATCACACGAGCA			
thal‐61	F: TCGAATTGTTATTCAACCCTATAGAA	(*rpl16‐rps3*) IGS, *rps3*	LSC	597
	R: AATCGATCTGATCCAGGTCATAA			
thal‐62	F: CCCTCGGTCTATTAGTGAACCA	*ycf2*	IR	562
	R: CCAAGCTCGAAGTACCATTTG			
thal‐64	F: ATATGCGCCCTCCACCTAC	*ndhF*	SSC	379
	R: TTTGATTGGTATGAATTTGTGAGAA			
thal‐65	F: ATGGATCCGACGAACAAAGT	*ndhF*	SSC	541
	R: GGCTCTTATGGGCGGTTTA			
thal‐68	F: TGTGTGGATCATTATTATCAGTAGCTC	*ccsA*	SSC	506
	R: TGAACCATAACTATGCAGCCCTA			
thal‐69	F: AAAGGTCTTACAAATCCAATACGC	(*ccsA‐ndhD*) IGS, *ndhD*	SSC	581
	R: CTCGATGGCTTCTCTTGCAT			
thal‐70	F: CCCAGAACTCCCATTAAGAGAA	*ndhD*	SSC	483
	R: TTTCCCTCATAGAGGAAATAAGGTT			
thal‐72	F: CCGATGGATAATAAATAGGCACTC	*ndhE*, (*ndhE‐ndhG*) IGS, *ndhG*	SSC	591
	R: TGTGATGTTCATCAATGGTTCA			
thal‐74	F: TCCGCTTAGCTTAACCCTTG	*ndhA*	SSC	525
	R: TCGTTTATTCAGTATCGGACCA			
thal‐75	F: AACACTCCGATCTCCTATCAGAA	*ndhH*	SSC	530
	R: GGATAGATAAATGTTTGGATTTCTGTG			
thal‐78	F: TGCGGCACTAATCTAGACCATC	*ycf1*	SSC	542
	R: TCCCGACTAATACGTAAATGTCAC			
thal‐80	F: TCTGAATACCGTCGATTAACCA	*ycf1*	SSC	503
	R: ATGCGTGCTCAAAGACGTAA			
thal‐81	F: CGTATCAAAGCCACTTCGTCT	*ycf1*	SSC	578
	R: CATCGCGGAACAATCAAA			

Note

IR = inverted repeat region; LSC = large single copy region; SSC = small single copy region.

a Primer pairs were designed for an annealing temperature of 60°C (±1°C). Validation consisted of successful (single amplicon) amplification on three test species and absence of (or minimal) primer dimer detection.

b Conserved sequence tags CS1 (5′‐ACACTGACGACATGGTTCTACA) and CS2 (5′‐TACGGTAGCAGAGACTTGGTCT) were added to each primer to make target‐specific primer for microfluidic PCR.

c Estimated from three *Thalictrum* species, including primer length.

**Figure 1:**
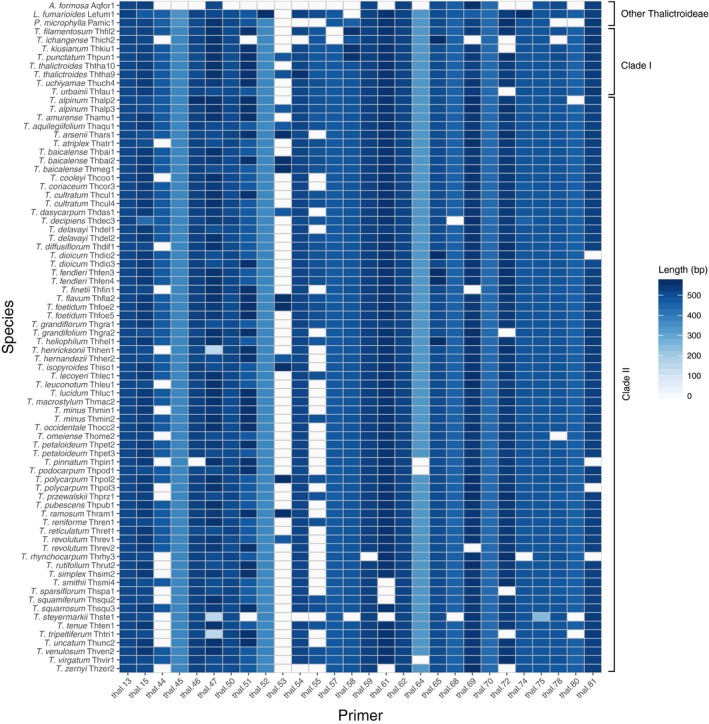
Cross‐amplification performance of chloroplast primers for phylogenetic studies in *Thalictrum*. Amplification results in 62 species of *Thalictrum* and one species of *Aquilegia*,* Leptopyrum*, and *Paraquilegia* (outgroups) with the 28 validated primer pairs. Darker shades of blue represent longer amplification products; white represents failed amplification. *Thalictrum* clades sensu Soza et al. (2012, 2013).

The cross‐amplification and sequencing resulted in regions with 15–75 (mean 69) consensus sequences of *Thalictrum*. Two regions, thal‐53 and thal‐55, had lower amplification success, with 15 and 40 sequences, respectively (Table 2, Fig. 1, Appendix S3). The amplification success per sample ranged from 19 to 28 (mean 26) regions. The amplification success in *Aquilegia*,* Leptopyrum*, and *Paraquilegia* was 12, 26, and 23 regions, respectively (Fig. 1), showing the potential utility of the newly developed primers on related genera in Thalictroideae. *Thalictrum‐*only alignment lengths ranged from 335 to 658 bp (mean 525 bp; Appendix S3), with the number of variable sites ranging from nine to 195 (mean 73). Alignments including the other Thalictroideae genera ranged from 335 to 725 bp (mean 544; Appendix S3) in length and contained 29–218 (mean 86) variable sites (Table 2). The total alignment length of the 28 regions (including all genera) was 15,268 bp, with 2443 variable sites and 92% character occupancy.

**Table 2:** Alignment summary statistics for 28 amplified chloroplast regions in *Thalictrum* and relatives.

Locus	*Thalictrum*					*Thalictrum* + *Aquilegia* + *Leptopyrum* + *Paraquilegia*				
	Alignment length, bp	No. of sequences	Sequence length range, bp (mean)	Pairwise identity, %	Variable sites, bp (PI)	Alignment length, bp	No. of sequences	Sequence length range, bp (mean)	Pairwise identity, %	Variable sites, bp (PI)
thal‐13	515	75	509–515 (509)	99.50	28 (12)	531	78	509–522 (509)	99.40	42 (14)
thal‐15	605	75	434–544 (525)	93.70	85 (36)	632	78	434–544 (524)	93.00	128 (45)
thal‐44	496	53	466–479 (473)	98.50	33 (20)	500	55	457–479 (472)	98.10	45 (22)
thal‐45	372	75	372–372 (372)	99.00	24 (15)	372	77	345–372 (371)	98.50	30 (15)
thal‐46	615	74	500–561 (521)	96.10	142 (34)	630	76	500–561 (520)	95.80	152 (43)
thal‐47	658	75	150–554 (518)	83.50	195 (140)	725	78	150–554 (518)	83.40	218 (150)
thal‐50	545	75	499–517 (506)	97.50	30 (14)	556	77	495–517 (506)	97.20	43 (14)
thal‐51	594	73	461–557 (512)	85.20	190 (140)	599	75	461–557 (511)	85.20	197 (142)
thal‐52	381	75	366–377 (370)	99.60	16 (11)	571	77	366–558 (372)	98.10	45 (11)
thal‐53	585	15	469–560 (520)	85.60	126 (78)	NA	NA	NA	NA	NA
thal‐54	567	72	505–558 (514)	98.30	76 (17)	575	73	505–558 (514)	98.20	78 (18)
thal‐55	504	40	474–495 (486)	97.30	28 (19)	524	41	474–501 (486)	97.10	32 (19)
thal‐57	495	73	464–480 (473)	99.00	36 (23)	496	75	464–480 (473)	98.90	45 (25)
thal‐58	568	74	482–556 (485)	98.10	39 (19)	573	75	458–556 (485)	97.90	48 (21)
thal‐59	524	74	518–524 (524)	99.40	33 (18)	524	77	518–524 (524)	99.20	53 (18)
thal‐61	590	70	532–553 (552)	96.10	153 (107)	591	72	532–553 (552)	96.00	165 (110)
thal‐62	519	75	519–519 (519)	99.80	9 (7)	525	78	519–525 (519)	99.70	29 (7)
thal‐64	335	72	335–335 (335)	98.50	50 (30)	335	74	335–335 (335)	98.40	58 (36)
thal‐65	579	75	496–563 (504)	96.80	77 (34)	586	78	496–563 (504)	96.70	93 (39)
thal‐68	456	73	447–456 (456)	98.90	47 (29)	474	76	447–468 (456)	98.60	71 (38)
thal‐69	616	72	521–558 (545)	96.00	96 (64)	620	75	462–558 (544)	95.30	111 (62)
thal‐70	436	75	436–436 (436)	99.20	38 (20)	436	78	436–436 (436)	99.00	52 (24)
thal‐72	658	68	530–556 (542)	97.10	66 (30)	662	70	530–556 (542)	96.70	103 (42)
thal‐74	495	74	454–488 (482)	98.40	38 (21)	593	76	454–560 (483)	97.60	50 (21)
thal‐75	488	75	249–480 (477)	97.70	104 (15)	486	78	249–480 (477)	97.80	82 (23)
thal‐78	508	73	469–502 (484)	98.10	54 (33)	556	75	469–502 (485)	97.50	83 (35)
thal‐80	460	72	454–460 (460)	98.10	105 (40)	460	73	454–460 (460)	97.90	118 (44)
thal‐81	545	71	502–545 (529)	97.40	121 (62)	551	74	502–545 (529)	97.00	146 (67)

Note

PI = parsimony informative; NA= not applicable.

**Figure 2:**
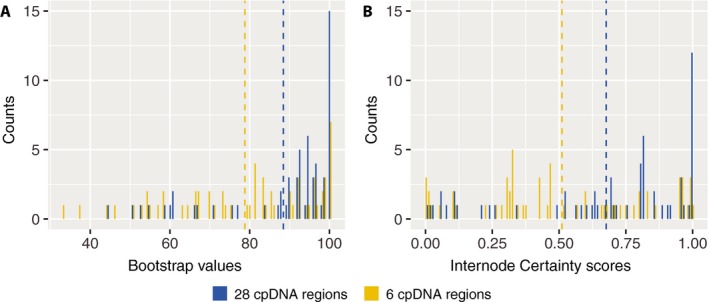
Overall performance of chloroplast primers for phylogenetic studies in *Thalictrum*. (A) Bootstrap value distribution of the 28‐region (blue) and six‐region (yellow) phylogenies. Dashed lines represent mean values. (B) Internode certainty scores distribution of the 28‐region (blue) and six‐region (yellow) phylogenies. Dashed lines represent median scores.

To test the usefulness of the newly generated chloroplast primers for improving phylogenetic resolution within *Thalictrum*, we inferred a phylogeny of 62 *Thalictrum* species (one individual per species) and three outgroups using all 28 regions and compared it to an inferred phylogeny of the same species using six chloroplast regions (*ndhA*,* rbcL*,* rpl16*,* rpl32‐trnL*,* trnL‐trnF*, and *trnV‐ndhC*) (Soza et al., 2012, 2013; Wang et al., 2018). For each concatenated matrix, we searched for the best partition scheme followed by maximum likelihood tree inference and 1000 ultrafast bootstrap replicates for node support using IQ‐Tree version 1.6.10 (Nguyen et al., 2014). As an additional measure of tree resolution, we estimated internode certainty scores (Salichos et al., 2014) using the majority rule consensus tree across 1000 bootstrap replicates in RaxML version 8.2.11 (Stamatakis, 2014). The six‐region matrix had an aligned length of 6650 bp and 363 parsimony‐informative sites, whereas the 28‐region matrix had an aligned length of 15,268 bp and 1045 parsimony‐informative sites. Mean bootstrap values of the 28‐region trees were higher than those of the six‐region trees (89% and 79%, respectively; Fig. 2A). Moreover, mean internode certainty scores were also higher in the 28‐region tree (0.68 and 0.51, respectively; Fig. 2B). In summary, these results show that the 28‐region chloroplast matrix produces a tree with overall higher node support than the six‐region matrix, and is therefore suitable for improved phylogenetic studies in *Thalictrum* and close relatives.

## CONCLUSIONS

Here, we contribute chloroplast primers for phylogenetic and comparative studies of *Thalictrum* and its close relatives in Thalictroideae. Furthermore, we demonstrate the utility of whole genome and transcriptome libraries as a source of chloroplast sequence data for PCR primer design. Out of the 81 chloroplast primer pairs reported here, 28 were successfully validated for use with the high‐throughput, Fluidigm‐based microfluidic PCR system. Finally, although this was not directly tested here, these primers could also be used for traditional PCR.

## Supporting information


**APPENDIX S1.** Characterization of the *Thalictrum thalictroides* plastome and comparison to the plastome of *T. coreanum*.

**APPENDIX S2.** Primer sequences of the *Thalictrum* chloroplast genome that failed to pass our validation criteria or were not validated.

**APPENDIX S3.** Length (in base pairs) of 28 amplified chloroplast regions in *Thalictrum* and relatives.

## Data Availability

The complete plastome of *T. thalictroides* and partial plastome of *T. hernandezii* were deposited in GenBank (MH092833 [WTBR], MH092834 [WT478], and MK716276). The alignment used for primer design (with primer and gene annotations), raw sequence data from the 28 amplified regions for the 78 samples of *Thalictrum* and relatives, alignments, and phylogenetic trees are available from the Dryad Digital Repository (https://doi.org/10.5061/dryad.hv4k73n; Morales‐Briones et al., 2019).

## APPENDIX 1 Source of genomic resources used for plastome assembly and annotation.

**Table:**

Species	Sample code	Usage	Accession no.	Notes
*Thalictrum hernandezii* Tausch ex J. Presl	WT441	Partial plastome assembly and primer design	SRA: SRR6869419, SRR6869420	Transcriptome libraries. Libraries represent staminate and hermaphrodite flowers from one andromonoecious individual.
*T. thalictroides* (L.) A. J. Eames & B. Boivin	WT478	Whole plastome assembly and primer design	SRA: SRR6869426, SRR6869425, SRR6869418	Genomic libraries. Each library has different insert size.
*T. thalictroides*	WTBR	Whole plastome assembly	SRA: SRR6869424, SRR6869423, SRR6869421	Genomic libraries. Each library has different insert size.
*T. coreanum* H. Lév.	—	Primer design and plastome annotation	GenBank: NC026103	
*Aconitum chiisanense* Nakai	—	Plastome annotation	GenBank: NC029829	
*Megaleranthis saniculifolia* Ohwi	—	Plastome annotation	GenBank: NC012615	
*Ranunculus macranthus* Scheele	—	Plastome annotation	GenBank: DQ359689	

## APPENDIX 2 Voucher information for species of *Thalictrum* used for primer validation and cross‐amplification.

**Table:**

Species	Sample code	Voucher (Herbarium)	Locality
*Aquilegia formosa* Fisch. ex DC.	Aqfor1	V. Di Stilio 128 (WTU)	Di Stilio Garden, Seattle, WA, USA
*Leptopyrum fumarioides* (L.) Rchb.	Lefum1	A. Liston 819‐13 (RSA)	Xinjiang, China
*Paraquilegia microphylla* (Royle) J. R. Drumm. & Hutch.	Pamic1	I. Smirnov 277 (RSA)	Irkutsk, Arshan, Russia
*Thalictrum alpinum* L.	Thalp2	D. E. Boufford et al. 32249 (F)	Xizang (Tibet), China
*Thalictrum alpinum* L.	Thalp3	V. Di Stilio 115 (WTU)	Cultivated from Ion Exchange Nursery, Iowa, USA
*Thalictrum amurense* Maxim.	Thamu1	Unvouchered	Cultivated at UC Botanical Garden at Berkeley, CA, USA
*Thalictrum aquilegiifolium* L.	Thaqu1	V. Di Stilio 108 (WTU)	Cultivated material from Cricklewood Nursery, CA, USA
*Thalictrum arsenii* B. Boivin	Thars1	A. Liston 1128 (OSC)	Mpio. Morelia, Jaripeo, Michoacan, Mexico
*Thalictrum atriplex* Finet & Gagnep.	Thatr1	D. E. Boufford et al. 32557 (GH)	Xizang (Tibet), China
*Thalictrum baicalense* Turcz.	Thbai1	Unvouchered	Cultivated at University of Washington Botany Greenhouse, Seattle, USA, from seeds from B & T World Seeds
*Thalictrum baicalense* Turcz.	Thbai2	R. Zhang 20120614‐01 (PE)	Ecological station, Dong Lin Mtn., China
*Thalictrum baicalense* Turcz.	Thmeg1	D. E. Boufford et al. 37958 (GH)	Sichuan, Danba Xian, China
*Thalictrum cooleyi* H. E. Ahles	Thcoo1	Unvouchered	The State Botanical Garden of Georgia, Athens, GA, USA
*Thalictrum coriaceum* (Britton) Small	Thcor3	A. Floden s.n. (TENN)	Greene Co., TN, USA
*Thalictrum cultratum* Wall.	Thcul1	D. E. Boufford et al. 31166 (F)	Xizang (Tibet), China
*Thalictrum cultratum* Wall.	Thcul4	D. E. Boufford et al. 31233 (GH)	Xizang (Tibet), China
*Thalictrum dasycarpum* Fisch. & Avé‐Lall.	Thdas1	V. Di Stilio 110 (WTU)	Cultivated from Ion Exchange Nursery, Iowa, USA
*Thalictrum decipiens* B. Boivin	Thdec3	L. Galetto 2089 (CORD)	Pampa de Achala, Córdoba, Argentina
*Thalictrum delavayi* Franch.	Thdel1	D. E. Boufford et al. 30452 (F)	Sichuan, Xiangcheng Xian, China
*Thalictrum delavayi* Franch.	Thdel2	V. Di Stilio 121 (WTU)	Cultivated from seed from B & T World Seeds, Aigues‐Vives, France
*Thalictrum diffusiflorum* C. Marquand & Airy Shaw	Thdif1	A. Liston 1161 (OSC)	Cultivated from Heronswood Nursery, Kingston, WA, USA
*Thalictrum dioicum* L.	Thdio3	M. Sain 60 (WIS)	University of Wisconsin campus, Muir Woods, Madison, WI, USA
*Thalictrum dioicum* L.	Thdio2	V. Di Stilio 101 (A)	Lithia Springs, South Hadley, MA, USA
*Thalictrum fendleri* Engelm. ex A. Gray	Thfen4	Unvouchered	Cultivated at UW Botany Greenhouse, Seattle, USA
*Thalictrum fendleri* Engelm. ex A. Gray	Thfen3	V. Soza 1920 (WTU)	Cultivated from Heronswood Nursery, Kingston, WA, USA
*Thalictrum filamentosum* Maxim.	Thfil2	V. Di Stilio 104 (WTU)	Cultivated from Heronswood Nursery, Kingston, WA, USA
*Thalictrum finetii* B. Boivin	Thfin1	D. E. Boufford et al. 33172 (GH)	Sichuan, Jiulong Xian, China
*Thalictrum flavum* L.	Thfla2	V. Di Stilio 109 (WTU)	Cultivated from Heronswood Nursery, Kingston, WA, USA
*Thalictrum foetidum* L.	Thfoe5	Unvouchered	Cultivated from Arrowhead Alpines nursery, Michigan, USA
*Thalictrum foetidum* L.	Thfoe2^a^	V. Soza 1923 (WTU)	Cultivated from Arrowhead Alpines nursery, Michigan, USA
*Thalictrum grandiflorum* Maxim.	Thgra1	Unvouchered	Cultivated from Heronswood Nursery, Kingston, WA, USA
*Thalictrum grandifolium* S. Watson	Thgra2	G. B. Hinton 20254 (TEX)	Coahuila, Mexico
*Thalictrum heliophilum* Wilken & DeMott	Thhel1	Mary Waters s.n. (CS)	Cathedral Bluffs, Rio Blanco County, CO, USA
*Thalictrum henricksonii* M. C. Johnst.	Thhen1	J. Henrickson 13417 (RSA)	Zacatecas, Mexico
*Thalictrum hernandezii* Tausch ex J. Presl	Thher2^a^	A. Liston 1125 (OSC)	Temascaltepec, State of Mexico, Mexico
*Thalictrum ichangense* Lecoy. ex Oliv.	Thich2	A. Floden 13116 (TENN)	Sapa, Lao Cai Province, Vietnam
*Thalictrum isopyroides* C. A. Mey.	Thiso1	V. Di Stilio 111 (WTU)	Cultivated from Heronswood Nursery, Kingston, WA, USA
*Thalictrum kiusianum* Nakai	Thkiu1	J. Brunet s.n. (OSC)	Cultivated at Corvallis, OR, USA
*Thalictrum lecoyeri* Franch.	Thlec1	D. E. Boufford et al. 37972 (GH)	Sichuan, Danba Xian, China
*Thalictrum leuconotum* Franch.	Thleu1	D. E. Boufford et al. 42102 (GH)	Yunnan, Zhongdian Xian, China
*Thalictrum lucidum* L.	Thluc1	V. Di Stilio 122 (WTU)	Cultivated from Arrowhead Alpines nursery, Michigan, USA
*Thalictrum macrostylum* Shuttlew. ex Small & A. Heller	Thmac2	Unvouchered	“Serpentine Barrens,” Chunky Gal Mountain, NC, USA
*Thalictrum minus* L.	Thmin1	H. W. Rickett & F. A. Stafleu 742 (OSC)	Gelderland, Netherlands
*Thalictrum minus* L.	Thmin2	V. Soza 1910 (WTU)	Cultivated at University of Washington Botany Greenhouse, Seattle, WA, USA
*Thalictrum occidentale* A. Gray	Thocc2	K. A. Beck 200712 (WTU)	Lower Boundary Dam Reservoir, Pend Oreille River, WA, USA
*Thalictrum omeiense* W. T. Wang & S. H. Wang	Thome2	A. Liston 1166 (OSC)	Cultivated from Heronswood Nursery, Kingston, WA, USA
*Thalictrum petaloideum* L.	Thpet3	Unvouchered	Dong Ling Mt., Betula Forest, Beijing, China
*Thalictrum petaloideum* L.	Thpet2	Unvouchered	Tong Ling Mt., Yin Ranje, Beijing, China
*Thalictrum pinnatum* S. Watson	Thpin1	R. M. Straw & M. Forman 1857 (RSA)	Chihuahua, Mexico
*Thalictrum podocarpum* Kunth ex DC.	Thpod1	M. Weigend 2000/623 (OSC)	Ancash, Huaylas, Pampanomas, Peru
*Thalictrum polycarpum* (Torr.) S. Watson	Thpol3	B. Keller s.n. (UC)	Cultivated at University of California Botanical Garden at Berkeley, CA, USA
*Thalictrum polycarpum* (Torr.) S. Watson	Thpol2	J. F. Smith 4572 (WTU)	Cassia County, Idaho, USA
*Thalictrum przewalskii* Maxim.	Thprz1	D. E. Boufford et al. 36521 (GH)	Sichuan, Dege Xian, China
*Thalictrum pubescens* Pursh	Thpub1	D. Baum & D. Howarth 375 (A)	Arnold Arboretum, Jamaica Plain, MA, USA
*Thalictrum punctatum* H. Lév.	Thpun1	V. Di Stilio 117 (WTU)	Cultivated from Heronswood Nursery, Kingston, WA, USA
*Thalictrum ramosum* B. Boivin	Thram1	A. Larsen s.n. (UC)	Cultivated at UC Botanical Garden at Berkeley, CA, USA
*Thalictrum reniforme* Wall.	Thren1	B. Keller s.n. (UC)	Cultivated at University of California Botanical Garden at Berkeley, CA, USA
*Thalictrum reticulatum* Franch.	Thret1	D. E. Boufford et al. 42802 (GH)	Sichuan, Muli Xian, China
*Thalictrum revolutum* DC.	Threv2	A. Floden 1347 (TENN)	Campbell County, TN, USA
*Thalictrum revolutum* DC.	Threv1	V. Soza 1917 (WTU)	Cultivated from USDA AMES #28275
*Thalictrum rhynchocarpum* Quart.‐Dill. & A. Rich.	Thrhy3	W. Kindeketa 820 (MO)	Arusha, Tanzania
*Thalictrum rutifolium* Hook. f. & Thomson	Thrut2	D. E. Boufford et al. 32127 (GH)	Xizang (Tibet), Riwoqe Xian, China
*Thalictrum simplex* L.	Thsim2	V. Soza 1914 (WTU)	Cultivated from USDA GRIN AMES #23805
*Thalictrum smithii* B. Boivin	Thsmi4	D. E. Boufford et al. 28205 (A)	Sichuan, Daocheng Xian, Gongling, China
*Thalictrum sparsiflorum* Turcz. ex Fisch. & C. A. Mey.	Thspa1	M. Williams 1630 (OSC)	7 miles from Seward, AK, USA
*Thalictrum squamiferum* Lecoy.	Thsqu2	D. E. Boufford et al. 32003 (GH)	Xizang (Tibet), Riwoqe Xian, China
*Thalictrum squarrosum* Stephan ex Willd.	Thsqu3	X. Duan 20120617 (PE)	Cultivated at CAS Botanical Garden, Beijing, China
*Thalictrum steyermarkii* Standl.	Thste1	T. B. Croat 40494 (MO)	Chiapas, Mexico
*Thalictrum tenue* Franch.	Thten1	W. Zhai 20120615 (PE)	Wanging, Henan, China
*Thalictrum thalictroides* (L.) A. J. Eames & B. Boivin	Ththa10^a^	V. Di Stilio 124 (WTU)	Cultivated from Arrowhead Alpines nursery, Michigan, USA
*Thalictrum thalictroides* (L.) A. J. Eames & B. Boivin	Ththa9	V. Di Stilio 123 (WTU)	Cultivated from natural population from Massachusetts, USA
*Thalictrum tripeltiferum* B. Boivin	Thtri1	L. E. Detling 8788 (ORE)	Jalisco, Mexico
*Thalictrum uchiyamae* Nakai	Thuch4	V. Di Stilio 113 (WTU)	Cultivated from seed from B & T World Seeds, Aigues‐Vives, France
*Thalictrum uncatum* Maxim.	Thunc2	D. E. Boufford et al. 30691 (GH)	Sichuan, Xiangcheng Xian, China
*Thalictrum urbainii* Hayata = *T. fauriei* Hayata	Thfau1	A. Liston 1162 (OSC)	Cultivated from Heronswood Nursery, Kingston, WA, USA
*Thalictrum venulosum* Trel.	Thven2	Voucher lost at OSC	NA
*Thalictrum virgatum* Hook. f. & Thomson	Thvir1	D. E. Boufford et al. 30496 (GH)	Sichuan, Xiangcheng Xian, China
*Thalictrum zernyi* Ulbr.	Thzer2	R. E. Gereau & C. J. Kayombo 3976 (MO)	Iringa, Ludewa, Tanzania

Note

NA = not available; s.n. = unnumbered.

a Sample used for primer validation and cross‐amplification.

## References

[aps311294-bib-0001] Boivin, B. 1944 American Thalictra and their old world allies. Contributions from the Gray Herbarium of Harvard University 152: 337–491.

[aps311294-bib-0002] Borowiec, M. L. 2016 AMAS: A fast tool for alignment manipulation and computing of summary statistics. PeerJ 4: e1660. 26835189 10.7717/peerj.1660 PMC4734057

[aps311294-bib-0003] Gitzendanner, M. A. , P. S. Soltis , T.‐S. Yi , D.‐Z. Li , and D. E. Soltis . 2018 Plastome phylogenetics: 30 years of inferences into plant evolution *In* Chaw S.‐M. and Jansen R. [eds.], Advances in Botanical Research, volume 85: Plastid genome evolution, 293–313. Academic Press, London, United Kingdom.

[aps311294-bib-0004] Jacobs, S. J. , C. Kristofferson , S. Uribe‐Convers , M. Latvis , and D. C. Tank . 2018 Incongruence in molecular species delimitation schemes: What to do when adding more data is difficult. Molecular Ecology 27: 2397–2413. 29701315 10.1111/mec.14590

[aps311294-bib-0005] Katoh, K. , and D. M. Standley . 2013 MAFFT Multiple Sequence Alignment Software Version 7: Improvements in performance and usability. Molecular Biology and Evolution 30: 772–780. 23329690 10.1093/molbev/mst010 PMC3603318

[aps311294-bib-0006] Kearse, M. , R. Moir , A. Wilson , S. Stones‐Havas , M. Cheung , S. Sturrock , S. Buxton , et al. 2012 Geneious Basic: An integrated and extendable desktop software platform for the organization and analysis of sequence data. Bioinformatics 28: 1647–1649. 22543367 10.1093/bioinformatics/bts199 PMC3371832

[aps311294-bib-0007] Liu, C. , L. Shi , Y. Zhu , H. Chen , J. Zhang , X. Lin , and X. Guan . 2012 CpGAVAS, an integrated web server for the annotation, visualization, analysis, and GenBank submission of completely sequenced chloroplast genome sequences. BMC Genomics 13: 715. 23256920 10.1186/1471-2164-13-715 PMC3543216

[aps311294-bib-0008] Lohse, M. , O. Drechsel , S. Kahlau , and R. Bock . 2013 OrganellarGenomeDRAW—a suite of tools for generating physical maps of plastid and mitochondrial genomes and visualizing expression data sets. Nucleic Acids Research 41: W575–W581. 23609545 10.1093/nar/gkt289 PMC3692101

[aps311294-bib-0009] Lowe, T. M. , and P. P. Chan . 2016 tRNAscan‐SE On‐line: Integrating search and context for analysis of transfer RNA genes. Nucleic Acids Research 44: W54–W57. 27174935 10.1093/nar/gkw413 PMC4987944

[aps311294-bib-0010] McKain, M. , and M. Wilson . 2017 mrmckain/Fast‐Plast: Fast‐Plast v.1.2.6 (Version v.1.2.6). Zenodo. 10.5281/zenodo.973887 [accessed 1 July 2017].

[aps311294-bib-0011] Morales‐Briones, D. F. , and D. C. Tank . 2019 Extensive allopolyploidy in the neotropical genus *Lachemilla* (Rosaceae) revealed by PCR‐based target enrichment of the nuclear ribosomal DNA cistron and plastid phylogenomics. American Journal of Botany 106: 415–437. 30882906 10.1002/ajb2.1253

[aps311294-bib-0012] Morales‐Briones, D. F. , T. Arias , V. S. Di Stilio , and D. C. Tank . 2019 Data from: Chloroplast primers for clade‐wide phylogenetic studies of *Thalictrum* . Dryad Digital Repository. 10.5061/dryad.hv4k73n. PMC6814179 31667022

[aps311294-bib-0013] Nguyen, L.‐T. , H. A. Schmidt , A. von Haeseler , and B. Q. Minh . 2014 IQ‐TREE: A fast and effective stochastic algorithm for estimating maximum‐likelihood phylogenies. Molecular Biology and Evolution 32: 268–274. 25371430 10.1093/molbev/msu300 PMC4271533

[aps311294-bib-0014] Park, S. , R. K. Jansen , and S. Park . 2015 Complete plastome sequence of *Thalictrum coreanum* (Ranunculaceae) and transfer of the *rpl32* gene to the nucleus in the ancestor of the subfamily Thalictroideae. BMC Plant Biology 15: 40. 25652741 10.1186/s12870-015-0432-6 PMC4329224

[aps311294-bib-0015] Salichos, L. , A. Stamatakis , and A. Rokas . 2014 Novel information theory–based measures for quantifying incongruence among phylogenetic trees. Molecular Biology and Evolution 31: 1261–1271. 24509691 10.1093/molbev/msu061

[aps311294-bib-0016] Shaw, J. , E. B. Lickey , E. E. Schilling , and R. L. Small . 2007 Comparison of whole chloroplast genome sequences to choose noncoding regions for phylogenetic studies in angiosperms: The tortoise and the hare III. American Journal of Botany 94: 275–288. 21636401 10.3732/ajb.94.3.275

[aps311294-bib-0017] Soza, V. L. , J. Brunet , A. Liston , P. S. Smith , and V. S. Di Stilio . 2012 Phylogenetic insights into the correlates of dioecy in meadow‐rues (*Thalictrum*, Ranunculaceae). Molecular Phylogenetics and Evolution 63: 180–192. 22289865 10.1016/j.ympev.2012.01.009

[aps311294-bib-0018] Soza, V. L. , K. L. Haworth , and V. S. Di Stilio . 2013 Timing and consequences of recurrent polyploidy in meadow‐rues (*Thalictrum*, Ranunculaceae). Molecular Biology and Evolution 30: 1940–1954. 23728793 10.1093/molbev/mst101

[aps311294-bib-0019] Stamatakis, A. 2014 RAxML version 8: A tool for phylogenetic analysis and post‐analysis of large phylogenies. Bioinformatics 30: 1312–1313. 24451623 10.1093/bioinformatics/btu033 PMC3998144

[aps311294-bib-0020] Straub, S. C. , M. Fishbein , T. Livshultz , Z. Foster , M. Parks , K. Weitemier , R. C. Cronn , and A. Liston . 2011 Building a model: Developing genomic resources for common milkweed (*Asclepias syriaca*) with low coverage genome sequencing. BMC Genomics 12: 211. 21542930 10.1186/1471-2164-12-211 PMC3116503

[aps311294-bib-0021] Tamura, M. 1995 Ranunculaceae *In* Hiepko P. [ed.], Die Natürlichen Pflanzenfamilien, 223–497. Duncker & Humblot, Berlin, Germany.

[aps311294-bib-0022] Twyford, A. D. , and R. W. Ness . 2016 Strategies for complete plastid genome sequencing. Molecular Ecology Resources 17: 858–868. 27790830 10.1111/1755-0998.12626 PMC6849563

[aps311294-bib-0023] Untergasser, A. , I. Cutcutache , T. Koressaar , J. Ye , B. C. Faircloth , M. Remm , and S. G. Rozen . 2012 Primer3—new capabilities and interfaces. Nucleic Acids Research 40: e115. 22730293 10.1093/nar/gks596 PMC3424584

[aps311294-bib-0024] Uribe‐Convers, S. , M. L. Settles , and D. C. Tank . 2016 A phylogenomic approach based on PCR target enrichment and high throughput sequencing: Resolving the diversity within the South American species of *Bartsia* L. (Orobanchaceae). PLoS ONE 11: e0148203. 26828929 10.1371/journal.pone.0148203 PMC4734709

[aps311294-bib-0025] Wang, T. N. , M. R. Clifford , J. Martínez‐Gómez , J. C. Johnson , J. A. Riffell , and V. S. Di Stilio . 2018 Scent matters: Differential contribution of scent to insect response in flowers with insect vs. wind pollination traits. Annals of Botany 123: 289–301. 10.1093/aob/mcy131 PMC6344221 30052759

