# Supplementary material for: Chloroplast primers for clade‐wide phylogenetic studies of Thalictrum
Source: Appl Plant Sci. 2019 Oct 16;7(10):e11294. doi: 10.1002/aps3.11294 (PMC6814179; doi:10.1002/aps3.11294)
Supplement: Supplementary file 1 — APPENDIX S1. Characterization of the Thalictrum thalictroides plastome and comparison to the plastome of T. coreanum. [file APS3-7-e11294-s001.docx]

**APPENDIX S1.** Characterization of the *Thalictrum thalictroides* plastome and comparison to the plastome of *T. coreanum*.

The plastome of *Thalictrum thalictroides* had a total length of 154,924 bp, comprising a large single copy (LSC; 84,899 bp), a small single copy (SSC; 17,479 bp), and two inverted repeat (IR) regions (IRA and IRB; 26,273 bp). The plastome encodes 130 genes (Tables S1, S2, Fig. S1), including 85 protein-coding, 37 tRNA, and eight rRNA genes. Seven protein-coding, seven tRNA, and four rRNA genes were duplicated in the IR region. *Thalictrum thalictroides* and *T. coreanum* are closely related (Park et al., 2015; both part of Clade I sensu Soza et al. (2012, 2013), and their chloroplast genomes are largely comparable (Table S1). The main differences are as follows: (1) The location of the small subunit ribosomal protein gene *rps19* is entirely in the LSC in *T. thalictroides*, while it is partially in the LSC and the IRA in *T. coreanum*; (2) A pseudogene copy of *rps19* is lacking from the IRB of *T. thalictroides*; and (3) The unknown gene *ycf15*, a widely conserved angiosperm gene that is transcribed but not translated (Schmitz-Linneweber et al., 2001; Raubeson et al., 2007; Shi et al., 2013), is present in the two IR copies of *T. thalictroides*. Similar to *T. coreanum*, the large subunit ribosomal protein gene *rpl32* and translational initiator gene *infA* were also missing in the plastome of *T. thalictroides* (pseudogene copies are present in the SSC and LSC, respectively); in *T. coreanum*, these have been functionally transferred to the nucleus (Park et al., 2015).

**LITERATURE CITED**

Park, S., R. K. Jansen, and S. Park. 2015. Complete plastome sequence of *Thalictrum coreanum* (Ranunculaceae) and transfer of the *rpl32* gene to the nucleus in the ancestor of the subfamily Thalictroideae. *BMC Plant Biology* 15: 40.

Raubeson, L. A., R. Peery, T. W. Chumley, C. Dziubek, H. M. Fourcade, J. L. Boore, and R. K. Jansen. 2007. Comparative chloroplast genomics: analyses including new sequences from the angiosperms *Nuphar advena* and *Ranunculus macranthus*. *BMC Genomics* 8: 174.

Schmitz-Linneweber, C., R. M. Maier, J.-P. Alcaraz, A. Cottet, R. G. Herrmann, and R. Mache. 2001. The plastid chromosome of spinach (*Spinacia oleracea*): Complete nucleotide sequence and gene organization. *Plant Molecular Biology* 45: 307–315.

Shi, C., Y. Liu, H. Huang, E.-H. Xia, H.-B. Zhang, and L.-Z. Gao. 2013. Contradiction between plastid gene transcription and function due to complex posttranscriptional splicing: An exemplary study of *ycf15* function and evolution in angiosperms. *PLOS ONE* 8: e59620.

**Table S1**. General features of the *Thalictum thalictroides* chloroplast genome and comparison to *T. coreanum.*

| **Chloroplast genome feature** | ***Thalictrum thalictroides*** | ***Thalictrum coreanum*^a^** |
| --- | --- | --- |
| Total size (bp) | 154,924 | 155,088 |
| LSC length (bp) | 84,899 | 84,733 |
| SSC length (bp) | 17,479 | 17,549 |
| IR length (bp) | 26,273 | 26,403 |
| Functional genes | 130 | 128 |
| Pseudogenes | 3 | 4 |
| Unique genes (i.e., excluding IR regions) | 114 | 113 |
| Protein-coding genes (duplicated in IR) | 78 (7) | 77 (6) |
| tRNA genes (duplicated in IR) | 30 (7) | 30 (7) |
| rRNA genes (duplicated in IR) | 4 (4) | 4 (4) |
| Genes with introns | 17 (7) | 17 (6) |
| Introns (duplicated in IR) | 20 (6) | 19 (5) |
| GC content, % | 38.4 | 38.4 |

*Note:* IR = inverted repeat region; LSC = large single copy region; SSC = small single copy region.

^a^ Park et al. (2015).

**Table S2.** Genes present in the *Thalictrum thalictroides* chloroplast genome.

| **Chloroplast genome feature** | **Gene products** |
| --- | --- |
| Photosystem I | *psaA, psaB, psaC, psaI, psaJ, ycf3*^f^*, ycf4* |
| Photosystem II | *psbA, psbB, psbC, psbD, psbE, psbF, psbH, psbI, psbJ, psbK, psbL, psbM, psbN, psbT, psbZ* |
| Cytochrome *b_6_/f* | *petA, petB*^e^*, petD*^e^*, petG, petL, petN* |
| ATP synthase | *atpA, atpB, atpE, atpF*^e^*, atpH, atpI* |
| RuBisCO | *rbcL* |
| NADH oxidoreductase | *ndhA*^e^*, ndhB*^a,e^*, ndhC, ndhD, ndhE, ndhF, ndhG, ndhH, ndhI, ndhJ, ndhK* |
| Large subunit ribosomal proteins | *rpl2*^a,e^*, rpl14, rpl16*^e^*, rpl20, rpl22, rpl23*^a^*, rpl32*^d^*, rpl33, rpl36* |
| Small subunit ribosomal proteins | *rps2, rps3, rps4, rps7*^a^*, rps8, rps11, rps12*^b,e^*, rps14, rps15, rps16*^e^*, rps18, rps19* |
| RNA polymerase | *rpoA, rpoB, rpoC1*^e^*, rpoC2* |
| Unknown function protein-coding gene | *ycf1*^c^*, ycf2*^a^*, ycf15*^a,e^ |
| Other genes | *accD, ccsA, cemA, clpP*^f^*, infA*^d^*, matK* |
| Ribosomal RNAs | *rrn4.5, rrn4.5, rrn5, rrn5, rrn16, rrn16, rrn23, rrn23* |
| Transfer RNAs | *trnA-UGC*^a,e^*, trnC-GCA, trnD-GUC, trnE-UUC, trnF-GAA, trnfM-CAU, trnG-GCC*^e^*, trnG-UCC, trnH-GUG, trnI-CAU*^a^*, trnI-GAU*^a,e^*, trnK-UUU*^e^*, trnL-CAA*^a^*, trnL-UAA*^e^*, trnL-UAG, trnM-CAU, trnN-GUU*^a^*, trnP-UGG, trnQ-UUG, trnR-ACG*^a^*, trnR-UCU, trnS-GCU, trnS-GGA, trnS-UGA, trnT-GGU, trnT-UGU, trnV-GAC*^a^*, trnV-UAC*^e^*, trnW-CCA, trnY-GUA* |

*Note:* IR = inverted repeat region; LSC = large single copy region; SSC = small single copy region.

^a^ Two gene copies in the IR.

^b^ Trans-splicing gene. One copy in LSC-IR(A) and one copy in LSC-IR(B).

^c^ One functional copy in the boundary between SSC and IR(B) and one pseudogene copy in the IR(A).

^d^ Only pseudogene copy present.

^e^ Gene containing a single intron.

^f^ Gene containing two introns.

**Figure S1.** Gene map of the *Thalictrum thalictroides* chloroplast genome. Genes drawn inside the circle are transcribed clockwise, while those drawn outside are transcribed counterclockwise. Different functional gene groups are color-coded. Variation in the GC content of the genome is shown in the middle circle. IR = inverted repeat region; LSC = large single copy region; SSC = small single copy region.
