# Supplementary material for: Chloroplast primers for clade‐wide phylogenetic studies of Thalictrum
Source: Appl Plant Sci. 2019 Oct 16;7(10):e11294. doi: 10.1002/aps3.11294 (PMC6814179; doi:10.1002/aps3.11294)
Supplement: Supplementary file 2 — APPENDIX S2. Primer sequences of the Thalictrum chloroplast genome that failed to pass our validation criteria or were not validated. [file APS3-7-e11294-s002.docx]

**APPENDIX S2.** Primer sequences of the *Thalictrum* chloroplast genome that failed to pass our validation criteria or were not validated.^a^

| **Locus** | **Primer sequences (5′–3′)^b^** | **Amplification region** | **Chloroplast region** | **Amplification length (bp)^c^** | **Validation status^d^** |
| --- | --- | --- | --- | --- | --- |
| thal-01 | F: CATGACTTATACGCTCGTGTCAAC  R: TGATTGGTTAGGAGACCGTGT | (*psbA-trnK-UUU*) IGS, *matK* | LSC | 384 | Failed |
| thal-02 | F: CACAAATATGGATTCCGATTCA  R: TATGCACTTGCTCACGATCC | *matK* | LSC | 597 | Failed |
| thal-03 | F: GGATCGTGAGCAAGTGCATA  R: CGAGCTTCTGTTCTTAATTTGAATAAT | *trnK-UUU* | LSC | 558 | Failed |
| thal-04 | F: AATCCGTTGCTTCATCCAAA  R: GGATTATCTTCAATTGGGTGGT | *trnK-UUU*, (*trnK-UUU-rps16*) IGS | LSC | 574 | Failed |
| thal-05 | F: TCGTGTAATTAACGCGAAGTTCT  R: TGTCTCAACAACTTGAGCAGAAT | *rps16* | LSC | 563 | Failed |
| thal-10 | F: AGGCGGAAAGAGAGGGATT  R: TCGTTTATTGAGAGGTAAAGGATTAAA | *trnS-GCU*, (*trnS-GCU–trnG-GCC*) IGS, *trnG-GCC* | LSC | 557 | Failed |
| thal-12 | F: TCGAGATCAGAAGCGAACTG  R: CATTACAATACCTCGCTCCGTA | *atpA* | LSC | 511 | Failed |
| thal-14 | F: AACTCGCACACACTCCCTTT  R: CCTTGGATTTGTTGCTTGATT | *atpF*, (*atpF-atpH*) IGS | LSC | 525 | Failed |
| thal-18 | F: AGGATTCGACAATGGGTTTG  R: GAATCGCCGAGGTATTTGTG | (*rpoC2-rpoC1*) IGS, *rpoC1* | LSC | 563 | Failed |
| thal-20 | F: TCTCAATTCGGGAGGAAGAA  R: ATGAATTAATGAAATGTTGGTTGG | *rpoC1* | LSC | 597 | Failed |
| thal-21 | F: GGAAACTTATAAAGTTCTTCCGTCA  R: GAACAGATCAAATACCGAATCCA | *rpoB*, (*rpoB-trnC-GCA*) IGS | LSC | 542 | Failed |
| thal-22 | F: TGGATTCGGTATTTGATCTGTTC  R: GTGTTTCCTTAATGGCATAGCA | (*rpoB-trnC-GCA*) IGS | LSC | 569 | Failed |
| thal-23 | F: GGGTCCAATTGAATTTGCAT  R: TCGCGAGTCTGTATACAAGGAA | (*rpoB-trnC-GCA*) IGS, *trnC-GCA*, (*trnC-GCA-petN*) IGS | LSC | 593 | Failed |
| thal-26 | F: ACATAAGAGTCAAAGTGGGCATT  R: GGATTCAAATTCGGCGTCT | (*petN-psbM*) IGS | LSC | 409 | Failed |
| thal-27 | F: CAAATCAAGAATCCCGTTGAA  R: TTCACCAGAGCACATACACAAA | (*psbM-trnD-GUC*) IGS | LSC | 573 | Failed |
| thal-28 | F: TGGACCTGACCCATTGAATC  R: CACCCACTCCATCAACTAATCTT | (*trnT-GGU-psbD*) IGS | LSC | 600 | Failed |
| thal-29 | F: GGGTTAAAGTACAACTTCTTGAATGA  R: GAGAGCTCCCTTTACCATTCTG | (*psbZ-trnG-GCC*) IGS, *trnG-GCC*, (*trnG-GCC–trnfM-CAU*) IGS, *trnfM-CAU*, (*trnfM-CAU-rps14*) IGS | LSC | 576 | Failed |
| thal-30 | F: TCACTAGAGCAATTATGGTCTGGA  R: AAGATCACGAAGCGATTTGAA | (*psaA-ycf3*) IGS, *ycf3* | LSC | 569 | Failed |
| thal-32 | F: TCTTCGCATTACCTCTCGTG  R: ATGACCCAGAAACCGAGTGA | (*ycf3-trnS-GGA*) IGS | LSC | 565 | Failed |
| thal-33 | F: GCCCTCGGTAACGAGACATA  R: GATGGTCATCGGTTCGATTC | *rps4*, (*rps4-trnT-UGU*) IGS, *trnT-UGU* | LSC | 471 | Failed |
| thal-37 | F: ACATAGATACACTCCTATGAATGTGGA  R: GGATCCCTTTGAACTTAGATTAGGA | *ndhC*, (*ndhC-trnV-UAC*) IGS | LSC | 536 | Failed |
| thal-38 | F: GGGATTCCTAATCTAAGTTCAAAGG  R: TGTTGGGTCTTTGAAACAGTTC | (*ndhC-trnV-UAC*) IGS, *trnV-UAC* | LSC | 547 | Failed |
| thal-39 | F: CCCTTAATTGTTTCTGTTAGACCAA  R: CCGGGATGTTAATGAACAAGA | *atpB* | LSC | 591 | Failed |
| thal-43 | F: TTACCGTGCGTTCTCTTAATTG  R: GGAATCGGGCTATCACTTCA | (*rbcL-accD*) IGS | LSC | 577 | Failed |
| thal-49 | F: AGCAGAAACCAAGGCTCTCA  R: TGTATGGGTAGATCTCATTGTGAAA | *rpl20*, (*rpl20-clpP*) IGS | LSC | 593 | Failed |
| thal-60 | F: ACGAACGGAAGCCCTTATTT  R: TCTTTCTGGGATCCGAATTG | *rpl36*, (*rpl36-infA*) IGS, *infA*, (*infA-rps8*) IGS | LSC | 523 | Failed |
| thal-63 | F: TTCGTCTCAGGTCGATGGAT  R: CCGAGTATCTGTTGATACGAAGTATT | *ycf5*, (*ycf15-trnL-CAA*) IGS | IR | 576 | Failed |
| thal-66 | F: AAATCAAATTAAAGAAGTTACGATTGG  R: AGAAAGAATGATTTATACCGGCTAGT | (*ndhF-rpl32*) IGS | SSC | 391 | Failed |
| thal-67 | F: CCGGTATAAATCATTCTTTCTTCG  R: AAGGGCTCAATTCAATGCAA | (*ndhF-rpl32*) IGS, *rpl32*, (*rpl32-trnL-UAG*) | SSC | 478 | Failed |
| thal-73 | F: CCCACATAAATAAGAAGCTGTGC  R: TCTTTCATTTCCTTGGTCAATAACT | *ndhG*, (*ndhG-ndhI*) IGS | SSC | 586 | Failed |
| thal-77 | F: TTTATTTGTTTATGAATCGCGAAA  R: GGTCTAGATTAGTGCCGCAAA | *ycf1* | SSC | 595 | Failed |
| thal-79 | F: CCTATCAGCCAGATCCCTCA  R: TGGTTAATCGACGGTATTCAGA | *ycf1* | SSC | 554 | Failed |
| thal-06 | F: TCCAGGATCGTTTATCCTTGTT  R: TCCTTGTTGAGACTTCGACAGA | *rps16*, (*rps16-trnQ-UUG*) IGS | LSC | 692 | Not validated |
| thal-07 | F: TCGAAGTCTCAACAAGGATTAATTT  R: CAAGATTGATTGTCCAGCATACC | (*rps16-trnQ-UUG*) IGS | LSC | 657 | Not validated |
| thal-08 | F: TCCATATTCCTATCCTACCTGTATCA  R: AACAAATGTTTCATTCTTGGAGATAA | (*rps16-trnQ-UUG*) IGS, *trnQ-UUG*, (*trnQ-UUG-psbK*) IGS | LSC | 640 | Not validated |
| thal-09 | F: AACAAATGTTTCATTCTTGGAGATAA  R: GGATTGCTAATCCGTTGTACG | (*psbK-psbI*) IGS, *psbI*, (*psbI-trnS-GCU*) IGS, *trnS-GCU* | LSC | 627 | Not validated |
| thal-11 | F: GTTCGATTCCCGCTACCC  R: GTTCGATTCCCGCTACCC | *trnG-GCC*, (*trnG-GCC–trnR-UCU*) IGS, (*trnR-UCU- atpA*) IGS, *atpA* | LSC | 609 | Not validated |
| thal-16 | F: TGAAGTCTTGTTTCCGTAGTGG  R: CGATACATCAGAACAATCATTTACAA | *rps2*, (*rps2-rpoC2*) IGS, *rpoC2* | LSC | 603 | Not validated |
| thal-17 | F: GCGTTATATCACCAGATCTCGATT  R: ACGTTGTTGAATAGAAATAAGGAATG | *rpoC2* | LSC | 643 | Not validated |
| thal-19 | F: CAAATAGCACGTCCTTCCACT  R: CCGAATTGAGACCGATCATT | *rpoC1* | LSC | 610 | Not validated |
| thal-24 | F: AGGGCTGGGCTATCAAGACT  R: ATTAAAGCAGCCCAAGCAAG | (*trnC-GCA-petN*) IGS, *petN* | LSC | 680 | Not validated |
| thal-25 | F: CAATGATTCCCATGCTCGTA  R: ACTTTGACTCTTATGTCTTACGGTTCT | (*petN-psbM*) IGS | LSC | 604 | Not validated |
| thal-31 | F: CCTCCCTTCTGTGCATAATGA  R: ACAGAGATGGTGCGATTTGA | *ycf3* | LSC | 646 | Not validated |
| thal-34 | F: GAAATTATGCAGTGGATAGGACTTT  R: GAGCCGTTTACGTTTAATAGATATGAT | (*trnT-UGU–trnL-UAA*) IGS | LSC | 638 | Not validated |
| thal-35 | F: GAGCCAAATCCTGGTTTCAG  R: TGGATGAGAAACATAACTAATGTGG | *trnL-UAA*, (*trnL-UAA–trnF-GAA*) IGS | LSC | 619 | Not validated |
| thal-36 | F: GTCCGCTTGCCTAGGACTC  R: TCTGGGCGTTTCTAATAATATCAAG | *ndhK*, (*ndhK-ndhC*) IGS, *ndhC* | LSC | 600 | Not validated |
| thal-40 | F: GATCAATTCCATGATGAGTACTGTTT  R: CCCACTTGCTATCGGACATT | *atpB*, (*atpB-rbcL*) IGS | LSC | 624 | Not validated |
| thal-41 | F: CATTTCGTTGTTTCTTATTTCATCA  R: TGTAGAAGATTCGGCAGCTACA | (*atpB-rbcL*) IGS, *rbcL* | LSC | 617 | Not validated |
| thal-42 | F: TGGGTAATGTATTTGGGTTCAA  R: CCCAAGGTGATCTCTCTTTCC | *rbcL* | LSC | 670 | Not validated |
| thal-48 | F: GCTCCACATATTCTTGTACAGTCAAA  R: AGAGCAACTCTTGAATTTGAATAAAGT | (*psbE-petL*) IGS | LSC | 610 | Not validated |
| thal-56 | F: TTAACTTGGGTTACGGGTGTG  R: GGGTTTACATGGTGGTTGGA | *petB*, (*petB-petD*) IGS, *petD* | LSC | 616 | Not validated |
| thal-71 | F: CATCGAATAACCTTATTTCCTCTATGA  R: TCAAAGTATCTTGTCCCTCTTTCA | *ndhD*, (*ndhD-psaC*) IGS, *psaC*, (*psaC-ndhE*) IGS | SSC | 629 | Not validated |
| thal-76 | F: CGCACCGATATCTGCCATA  R: CGGCTACTGGCTTATTTGTCA | *ndhH*, (*ndhH- rps15*) IGS, *rps15* | SSC | 620 | Not validated |

*Note:* IR = inverted repeat region; LSC = large single copy; SSC = small single copy.

^a^ Primer pairs were designed for an annealing temperature of 60°C (±1°C). Validation consisted of successful (single amplicon) amplification on three test species and absence of (or minimal) primer dimer detection.

^b^ Conserved sequence tags CS1 (5′-ACACTGACGACATGGTTCTACA) and CS2 (5′-TACGGTAGCAGAGACTTGGTCT) were added to each primer to make target-specific primer for microfluidic PCR.

^c^ Estimated from three *Thalictrum* species, including primer length.

^d^ Failed primers for this study are listed for their potential use under more permissive annealing temperatures and non-validated ones as a source of additional primers.
